# A Study of the Genus *Limnophora* Robineau-Desvoidy (Diptera: Muscidae), with Descriptions of Six new species from China

**DOI:** 10.1673/031.012.1701

**Published:** 2012-02-06

**Authors:** Wan-Qi Xue, Shu-Chong Bai, Wen-Xiu Dong

**Affiliations:** ^1^ Institute of Entomology, Shenyang Normal University, Huanghe North Street 253, Huanggu District, Shenyang 110034, China; ^2^ Institute of Paleontological, Shenyang Normal University, Huanghe North Street 253, Huanggu District, Shenyang 110034, China

**Keywords:** China, Diptera, Muscidae, *Limnophora*, key, new species, new record

## Abstract

This paper provides the characters of genus *Limnophora* (Diptera: Muscidae) and a key to the Chinese species of *Limnophora,* six new species collected from Hainan Island of China, namely, *L. brevispatula*, **n. sp.**, *L. cothurnosurstyla*, **n. sp.**, *L. dyadocerca*, **n. sp.**, *L. longitarsis*, **n. sp.**, *L. nuditibia*, **n. sp.** and *L. ypocerca*, **n. sp.** are diagnosed, described and illustrated, a new record species in China, namely, *L. argentifrons* (Shinonaga *et* Kano, 1977), is also included.

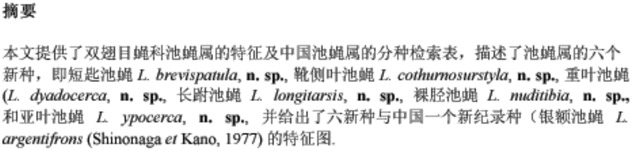

## Introduction

*Limnophora* Robineau-Desvoidy is a large genus of Muscidae (Diptera). RobineauDesvoidy established the genus in 1830, with *L. palustria* as the type species. This genus has a worldwide distribution, according to recent data, there are approximately 230 species in the world, including 66 species and a subspecies in China. In recent years, many scholars collected Muscidae specimens from Hainan Island of China, including six new species and a new record species belong to genus *Limnophora.* Hainan Island belongs to Oriental regions, and located on the northwest of the South China (18°10′–20°9′N, 108°36′–112°2′E), it with a tropical moist monsoonal climate, and has many mountain ranges, valleys, streams and rivers, the various environments and special climate are fit for *Limnophora* species to live.

## Materials and Methods

Specimens were collected from Hainan Island of China. They were examined using an Olympus SZ-ST stereomicroscope (www.olympus.com): further details (dissection, drawing, etc.) were studied under an Motic SMZ-140 dissecting stereomicroscopes. Male and female genitalia were examined and illustrated after they were dissected from the fly's bodies, all illustrations were drawn on ink jet plotter paper. The genitalia of dissected specimens were mounted in glycerol in a plastic tube on the staging pin.

The morphological terminology follows that of McAlpine (1981). Absolute measurements were used for the body length in millimeters. Descriptions of a species are done in the following order: body length, head, thorax, wing, legs and abdomen. The type specimens of the new species and examined specimens described herein are deposited in IESNU and IZCAS.

**Abbreviations.** — ***ori*,** frontal setae; ***ors*,** orbital setae; ***acr*,** acrostichal setae; ***prst-acr*,** presutural acrostichal setae; ***prst-dc*,** presutural dorsocentral setae; ***dc*,** dorsocentral setae; ***ia*,** intra-alar setae, ***pra*,** prealar setae; ***av*,** anteroventral setae; ***ad*,** anterodorsal setae; ***pd*,** posterodorsal setae; ***pv*,** posteroventral setae; ***d*,** dorsal setae; ***p*** posterior setae; **Co.** Collected; **IESNU** Institute of Entomology, Shenyang Normal University, China; **IZCA S** (Institute of Zoology, Chinese Academy of Sciences, China

**Genus *Limnophora* Robineau-Desvoidy, 1830 (Diptera: Muscidae)**
*Limnophora* Robineau-Desvoidy, 1830: 517. Type-species: *Limnophora palustria* Robineau-Desvoidy, 1830 (= *Limnophora* maculosa (Meigen, 1826)).
Coquillet Robineau-Desvoidy 1830; *Leucomelina* Macquart 1851; *Microchylum.*
Macquart 1851; *Melanochelia* Rondani 1866; *Pseudolimnophora* Strobl 1893; *Calliophrys* Kowarz 1893; *Limnophorites* Schnabl *et* Dziedzicki 1911; *Bucephalomyia* Malloch 1918; *Onychomyia* Stein 1919; *Emmesina* Malloch 1921; *Limnina* Malloch, 1928; *Apisia* Séguy, 1950.
**Identified Characteristic. --** Basisternum of prosternum with hairs, anepimeron and meron all bare; dorsal and ventral of radial node with small setae, R_4+5_ vein with hairs in a few specimens; hind tibia without *pd*, and usually without apical *ad,* if with small *ad,* the length less than the hind tibia's diameter; tergites 3 and 4 always with 1 pair of triangular blackish-brown spots; body length 1.5–5.0 mm.
**Distribution.** -- All over the world.

**Key to the Chinese species of genus *Limnophora* Robineau-Desvoidy (♂♂)**
1 Sternite 1 bare 2
- Sternite 1 with small hairs 66
2 *Dc* 2+3 3
- *Dc* 2+4 40
3 Frons about 1/4 of head in width at least 4
- Frons about 1/7 of head in width at most 22
4 Frons with 1 pair of *ors* 5
- Frons with 2 pairs of *ors* 8
5 Parafacial subequal with the width of postpedicel, fronto-orbital plate and parafacial covered with argentate pruinosity *L. pollinifrons* Stein, 1916
- Parafacial line-shaped, about 1/2 of postpedicel in width at most, fronto-orbital plate and parafacial covered with brown pruinosity 6
6 Mid femur with 1 *av,* mid tibia with 2 *p* *L. rufimana* (Strobl, 1893)
- Mid femur without *av,* mid tibia with 1*p* 7
7 Frontal vitta about 8.0 times as wide as fronto-orbital plate; mid femur with 1 *pd* ***L. dyadocerca* Xue, Bai *et* Dong, n. sp.**

- Frontal vitta about 3.5 times as wide as fronto-orbital plate; mid femur with 2 *pd* ***L. subcerca* Xue, Bai *et* Dong, n. sp.**

8 1st and 2nd tarsomeres of mid legs white *L. albitarsis* Stein, 1915
- All tarsomers of mid legs black 9
9 Dorsal and ventral surface of R_4+5_ vein with small hairs *L. veniseta* Stein, 1915
- Only basal part of R_4+5_ vein with hairs 10
10 Hind tibia without *av* ***L. nuditibia* Xue, Bai *et* Dong, n. sp.**

- Hind tibia with *av* 11
11 Fronto-orbital plate and parafacial all covered with argentate white pruinosity *L. argentifrons* (Shinonaga *et* Kano, 1977) **n. record in China**

- Fronto-orbital plate and parafacial covered with different colors pruinosity 12
12 Frontal vitta and fronto-orbital plate covered with black pruinosity, lower half of parafacial wirelike *L. beckeri* Stein, 1908
- Frontal vitta and fronto-orbital plate covered with different colors pruinosity, parafacial broad and not line-shaped 13
13 Parafacial about 3/10 width of postpedicel at most 14
- Parafacial about 1/2 width of postpedicel at least 15
14 Frontal vitta about 3.0 times as wide as fronto-orbital plate, pretarsus about 1.6 times as long as fore tibia *L. daduhea* Feng, 2001
- Frontal vitta about 6.0 times as wide as fronto-orbital plate, pretarsus subequal with fore tibia in length *L. nigrisquama* Tong, Xue *et* Wang, 2004
15 Vibrissal angle situated behind frontal angle in profile, frontal angle become obtuse angle, parafacial about half of postpedicel 16
- Vibrissal angle front of frontal angle in profile, frontal angle almost become right angle, parafacial subequal with postpedicel in width 21
16 Scutum with distinct strip, basal half of scutellum covered with blackish-brown pruinosity *L. pubiseta* Emden, 1965
- Scutum without distinct strip, basal half of scutellum without obvious blackish-brown pruinosity 17
17 Frontal vitta greyish-white 18
- Frontal vitta blackish-brown 19
18 Calypters greyish-white; apical of surstyli intumescent in profile *L. argentata* Emden, 1965
- Calypters yellowish; apical of surstyli not intumescent in profile *L. yulongxueshanna* Xue *et* Tong, 2003
19 Lower calypter brown; scutum without vitta; mid tibia with 2 *p,* 1 basal *pv* and 1 medial *pv* *L. papulicerca pubertiseta* Xue *et* Zhang, 1995
- Lower calypter white or yellowish; scutum with 3 dark brown vittae; mid tibia with 1 *p,* 1 basal *pv* 20
20 Scutum without vitta; surstyli thin and long, apical half of surstyli curved as hook-like in profile *L. triangula* (Fallén, 1825)
- Scutum with 3 brown black vittae; surstyli wide and short, apical half of surstyli not curved in profile *L. papulicerca* Xue *et* Zhang, 1998
21 Frontal triangle covered with argentate pruinosity, extending to the anterior margin of frons, postpedicel about 3.0 times as long as wide; with 2 rows of setiform *prst-acr* *L. argentitriangula* Xue *et* Wang, 1985
- Frontal triangle covered with gray pruinosity, not extending to the anterior arginal of frons, postpedicel about 2.0 times as long as wide; with 4 rows of trichoid *prstacr* *L. cyclocerca* Zhou *et* Xue, 1987
22 Frons wider than the postpedicel in width 23
- Frons equal to or narrower than the postpedicel in width 31
23 Presutural area of scutum without blackish-brown spots 24
- Presutural area of scutum with 3 blackish-brown spots 26
24 Calypters grayish-white; hind femur with 3–4 *pv* *L. albonigra* Emden, 1965
- Calypters brown; hind femur without *pv* 25
25 Thorax with 4 rows of trichoid *prst-acr,* katepisternal setae 1+2; fore tarsus subequal with fore tibia in length ***L. brevispatula* Xue, Bai *et* Dong, n. sp.**

- Thorax with 2–3 rows of trichoid *prst-acr,* katepisternal setae 1+1; fore tarsus about 1.8–1.9 times as long as fore tibia ***L. longitarsis* Xue, Bai *et* Dong, n. sp.**

26 Fore tibia with *pv* indistinctly; the black spot of tergite 4 subequal with the the tergite in length 27
- Fore tibia with *pv* distinctly; the black spot of tergite 4 about half of the tergite in length 28
27 *Ia* 0+2; mid tibia with 2 *p,* basal of scutellum without black transverse stripe *L. emeishanica* Feng, 2005
- *Ia* 0+1; mid tibia with 1 *p*, basal of scutellum with 1 black transverse stripe *L. fallax Stem,* 1920
28 Frontal vitta about half of fronto-orbital plate in width; thorax with 2 rows of *prst-acr;* mid tibia with 1 *p* *L. mongolica* Xue *et* Zhang, 1997
- Frontal vitta about 1.0–2.5 times as wide as fronto-orbital plate; thorax with 3–4 rows of *prst-acr;* mid tibia with 2–3 *p* 29
29 Thorax with 3 rows of *prst-acr*; tergite 5 brownish-yellow entirely, distal part with 1 pair of long setae *L. cinerifulva* Feng, 1999
- Thorax with 4 rows of *prst-acr*; tergite 5 with 1 pair of brown spots, and without long seta 30
30 Distal of sternite 5 closed to quadrate in profile, inner branch of surstyli long, and with setae *L. septentrionalis* Xue, 1984
- Distal of sternite 5 pointed in profile, inner branch of surstyli short, and without any hairs *L. minutifallax* Lin *et* Xue, 1986
31 Apical of the surstyli long spoon-shaped *L. longispatula* Xue *et* Tong, 2003
- Apical of the surstyli not long spoon-shaped 32
32 Tergite 5 with spot 33
- Tergite 5 without spot *L. oreosoacra* Feng, 2005
33 Tergite 5 with 1 spot or 2 indistinct spots, if with 2 spots, scutellum black or almost, apical of scutellum without lightly pruinosity 34
- Tergite 5 with 2 light brown spots obviously, apical half of scutellum covered with grey pruinosity 38
34 Dorsal and ventral surface of R_4+5_ vein with small seta rows 35
- Only basal part of R_4+5_ vein with hairs 36
35 Frons with 3 pairs of proclinate *ors*; thorax with 1 prealar seta; posterior margin of syntergite 1+2 with 1 pair of indistinct black spots -*L. yunnanensis* Xue et Tong, 2003
- Frons with 4 pairs of proclinate *ors;* thorax without prealar seta; syntergite 1+2 without black spot *L. spoliata* Stein, 1915
36 Frons with 3 pairs of *ors,* 2 pairs of *ori*; fore tibia with 1 medial *p*, mid and hind tibiae brown *L. brunneitibia*

*-* Frons with 1–2 pairs of *ors,* 4 pairs of *ori* at least; fore tibia without medial *p,* mid and hind tibiae black 37
37 Tergite 5 with 1 indistinct triangular spot, lateral lobe of sternite 5 tapering in profile, distal of cerci intumescent *L. nigra Xue*, 1984
- Tergite 5 with 1 distinct pair triangular brown spots, lateral lobe of sternite 5 round distally in profile, distal of cerci intumescent *L. prominens* Stein, 1904
38 Lateral lobe of sternite 5 round distally in profile, tergite 5 with 1 median vitta between 2 spots *L. nigrilineata* Xue, 1984
- Lateral lobe of sternite 5 tapering in profile, tergite 5 without median vitta between 2 spots 39
39 The median strip scutum not reaching to scutoscutellar suture; sternite 5 flat, its lateral lobe tapering *L. parastylata* Xue, 1984
- The median strip scutum reaching to scutoscutellar suture; sternite 5 not flat, its lateral lobe wide *L. purgata* Xue, 1992 40 Anterior half of postscutum with brownish-black transverse stripe-----41
- Anterior half of postscutum without brownish-black transverse stripe 47
41 Frons about 3.5 times of as wide as postpedicel at least; sternite 2 with strong setae, sternite 5 with dense and short setae cluster *L. exigua* (Wiedemann, 1830)
- Frons 2.5 times as wide as postpedicel at most, sternites 2 and 5 without above setae 42
42 Frontal vitta about 4.0 times as wide as fronto-orbital plate 43
- Frontal vitta abot 2.0 times as wide as fronto-orbital plate at most 45
43 Katepisternal setae 1+1; hind fumur without *av* *L. surrecticerca* Xue *et* Zhang, 1997
- Katepisternal setae 1+2; hind fumur with 2 *av* at least 44
44 Frons with 8–9 pairs of *ori*; mid fumur without *pv*, hind tibia with 2–4 *av* *L. furcicerca* Xue *et* Liu, 1990
- Frons with 4 pairs of *ori*; mid fumur with 2 *pv*, hind tibia without *av* ***L. cothurnosurstyla* Xue, Bai *et* Dong, n. sp.**

45 Between the brownish-black transverse stripe of postscutum and scutellum with with a narrow greyish-brown vitta *L. tigrina* (Am Stein, 1860)
- Between the brownish-black transverse stripe of postscutum and scutellum without greyish-brown vitta 46
46 Frons narrower than eye in width distinctly, fronto-orbital plate covered with greyish-white to brown pruinosity *L. himalayensis* Brunetti, 1907
- Frons subequal with eye in width, Fronto-orbital plate covered with silvery white pruinosity *L. suturalis* Stein, 1915
47 Frons about 1/4 of head in width at least, about 3.5 times as wide as postpedicel 48
- Frons about 1/6 of head in width at most, about 2.5 times as wide as postpedicel 51
48 Katepisternal setae 1+2 49
- Katepisternal setae 1+1 50
49 Fronto-orbital plate covered with dense and brown pruinosity, arista short plumose *L. apiciseta* Xue et Zhang, 1998
- Fronto-orbital plate covered with grey pruinosity, arista pubescent *L. nigripes*

50 Fronto-orbital plate covered with brown pruinosity; tergites 2 and 3 without triangular lateral spots *L. breviventris* Stein, 1915
- Fronto-orbital plate covered with grey pruinosity; tergites 2 and 3 with triangular lateral spots *L. flavifrons* Stein, 1915
51 Hind fumur with *pv* rows; distal of cerci with forcipate-like process 52
- Hind femur without *pv* row; distal of cerci without forcipate-like process 53
52 Epistoma projecting; R_4+5_ vein with small seta; mid and hind femora with 1 entire *pv* row separately *L. setinerva* Schnabl, 1911
- Epistoma not projecting; R_4+5_ vein without small seta; only basal half of mid femur with *pv,* and basal 3/5 of hind femur with *pv* *L. biprominens* Zhang *et* Xue, 1996
53 Frons only about 1/15 of head in width, and subequal with anterior ocellus in width; hind femur with entire *av* row *L. virago* Emden, 1965
- Frons about 1/10 of head in width at least, and about 1.5 times as wide as anterior ocellus; only apical half of hind femur with *av* row 54
54 Eye holoptic; mid femur only with 1 long and strong medial *a* *L. conica* Stein, 1915
- Eye dichoptic; basal half of mid femur with 1 *a* row at least 55
55 Calypter light brown to brown, fronto-orbital plate covered with brown or brown grey pruinosity 56
- Calypter white, if yellowish, fronto-orbital plate covered with white or silvery grey pruinosity normally 57
56 Parafacial about 1.3 times as wide as postpedicel; thorax with 4 rows of *prst-acr*; mid femur with 2 *pd,* 3–4 *pv,* hind tibia with 2 *av* *L. leptosternita* Tong, Xue *et* Wang, 2004
- Parafacial subequal with postpedicel; thorax with 2 rows of *prst-acr*; mid femur without *pd* and *pv*, hind tibia without *av* *L. brunneisquamam* Mu *et* Zhang 1990
57 Frontal vitta about 2.5 times as wide as fronto-orbital plate; mid tibia with 3 *p* *L. reventa* Feng, 1999
- Frontal vitta equal to or narrower than fronto-orbital plate in width; mid tibia with 2 *p* 58
58 Frontal vitta not line-shaped, frons about 1.5–2.0 times as wide as the distance of outer margin of posterior ocellar 59
- Frontal vitta line-shaped, frons equal to or narrower than the distance of outer margin of posterior ocellar in width 65
59 Fronto-orbital plate about 2.3 times as wide as frontal vitta; mid femur with 1 apical *pd* *L. apicicerca* Xiang *et* Xue, 1998
- Fronto-orbital plate about 1.5 times as wide as frontal vitta at most; mid femur with 2 apical *pd* 60
60 Thorax with 4 rows of *prst-acr,* without *pra* 61
- Thorax with 2 rows of *prst-ac, pra* hair-like 62
61 Fronto-orbital plate covered with silvery white pruinosity; katepisternal setae 1+1 *L. tibetan* Xue et Zhang, 1998
- Fronto-orbital plate covered with brownish-grey pruinosity; katepisternal setae 1+2 *L. nigriscrupulosa* Xiang *et* Xue, 1998
62 Frontal angle situated behind vibrissal angle in profile; mid tibia with 1 *p* *L. asiatica Xue et* Zhang, 1998
- Vibrissal angle situated behind frontal angle in profile; mid tibia with *2 p* 63
63 *Pra* 2, scutum covered with dark gray pruinosity *L. scrupulosa* (Zetterstedt, 1845)
- *Pra* 1, scutum covered with light gray pruinosity 64
64 Katepisternal setae 1+2, posterior half of tergite 5 with 1 brown triangular median vitta; basal half of surstyli wide, which becoming narrow toward to apical in profile *L. subscrupulosa* Zhang *et* Xue, 1990
- Katepisternal setae 1+1, tergite 5 with 1 brown median stripe; apical of surstyli wide and narrow in middle part in profile *L. orbitalis* Stein, 1907
65 Frons with 2 pairs of proclinate *ors*; tergite 5 with brown median vittae *L. interfrons* Xue, 1982
- Frons with 1 pair of proclinate *ors*; tergite 5 without median vitta. *L. latiorbitalis*
66 Thorax with 4 rows of *prst-acr,* katepisternal setae 1+1 *L. bannaensis* Zhang, Xue *et* Wang, 1998
- Thorax with 2 rows of *prst-acr*, katepisternal setae 1+2 *L. latifrons* Zhang *et* Xue, 1996

**Figure 1.**
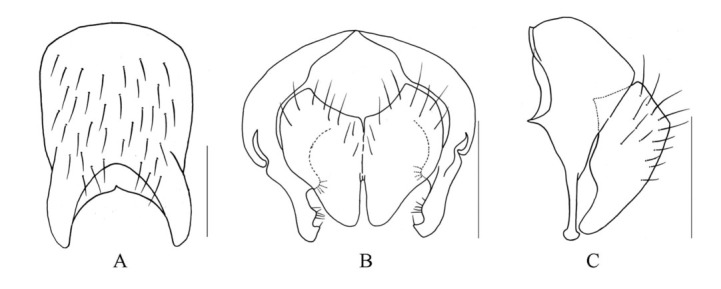
*L. argentifrons* (Shinonaga et Kano, 1977). A. male, sternite 5 in ventral view; B. male, Cerci and surstyli in posterior view; C. male, Cerci and surstyli in profile. Scale = 0.2 mm. High quality figures are available online.

***Limnophora argentifrons* (Shinonaga *et* Kano, 1977), n. record in China  (Figure 1A–C)**
**Material examined.** -- China, Hainan Island, Mt. Jianfeng, 700–800 m, 29 April 2008, Shu-Chong Bai Co., 2 ♂♂ (IESNU), 1 ♂ (IZCAS); 30 April 2008, Shu-Chong Bai and Xu-Dong Fei Co., 2 ♂♂ (IESNU), 2 ♂♂ (IZCAS).
**Distribution.** -- Japan, Ryukyu Islands (Iriomote Island, Okinawa main Island and Amamioshima Island); China, Hainan Island (Mt. Jianfeng).

**Figure 2.**
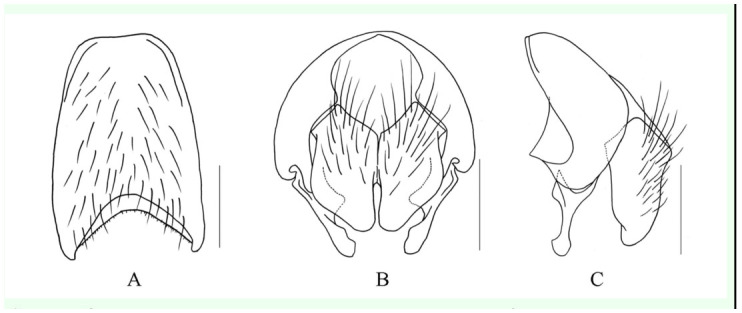
*L. brevispatula* Xue, Bai et Dong, n. sp.. A. male, sternite 5 in ventral view; B. male, Cerci and surstyli in posterior view; C. male, Cerci and surstyli in profile. Scale = 0.2 mm. High quality figures are available online.

***Limnophora brevispatula* Xue, Bai *et* Dong, n. sp.  (Figure 2A–C)**
**Holotype male.** -- Body length 4.8–5.0 mm.
***Head*.** Eye bare; frons about 1.2 times as wide as postpedicel, about 1/10 of head in width, lower 3/5 of frons with 5–6 pairs of intilted *ori,* upper 2/5 with 3 pairs of thin *ors,* frontal vitta black, about 1.5–2.0 times as wide as fronto-orbital plate, ocellar seta longer than the longest *ori,* fronto-orbital plate and parafacial covered with gray white pruinosity, parafacial about 2/5 of postpedicel in width; antennae black, postpedicel about 3.0 times as long as wide, arista short ciliated, the longest hair shorter than aristal basal diameter, lunule brown, frontal angle situated behind vibrissal angle in profile; gena about 1/8 of eye in height, genal and postgena hairs all black, the upper lateral area of the occiput with black hairs, prementum shining, about 3.0 times as long as wide; labella small, the length subequal with prementum height, palpus black, slightly longer than prementum.
***Thorax*.** Black in ground color, proepisternum covered with gray pruinosity distinctly, scutum black mostly, lateral of anterior of transverse suture to notopleuron covered with gray pruinosity, anterior part of transverse suture covered with gray pruinosity, anterior 3/4 of postscutum with black transverse stripe, posterior margin of transverse stripe extending to back, but not reaching to scutoscutellar suture; 4 rows of *trichoid prst-acr,* only 1 pair distinct before scutellum, *dc* 2+3, *ia* 0+1, *pra* hair-like; scutellum all black, lateral and ventral surface bare; basisternum of prosternum with hairs, notopleural, proepisternum hollow in centre, anepimeron, meron and katepimeron all bare; proepisternal setae 2, upper anterior anepisternal seta 1, katepisternal setae 1+2, the lower one only about 1/3 of the upper one.
***Wing*.** Transparent slightly, veins brown, basicosta black, costal spine small and short, distal of Sc slightly straight, dorsal and ventral surface of R_4+5_ vein in basal part and rascial node all with setae, calypters light brown, its margin brown, lower calypter protrude to tongue-shaped; halter yellow.
***Legs*.** Entirely black; fore tibia without median *p*; mid femur without *av,* basal 2/5 with setiform *a,* the super-medial one strong, 2 apical *pd,* with 2 *pv,* mid tibia with 1 median *p;* hind femur with 2 *av* in apical 1/3, without *pv,* hind tibia with 1 median *av,* 1 supermedial *ad,* without *pv*; mid and hind tarsi longer than tibia, claw and pulvilli short and small, about half of 5th tarsomere in length.
***Abdomen*.** Black in ground color, oviformshaped, covered with brownish-gray pruinosity, tergites 3 and 4 with a pair of triangulare lateral spots respectively, the widest part of spot wider than the length of tergite, tergite 5 with a trapeziform median spot indistinctly, syntergite 1+2 black except lateral of posterior part with brownish-grey pruinosity, sternite 1 bare. Tternite 5, cerci and surstyli as in Figure 2A–C.
**Female.** -- Unknown.
**Type material.** -- ***Holotype*.** China, Hainan Island, Mt. Jianfeng, 700–800 m, 30 April 2008, Shu-Chong Bai Co., ♂ (IESNU). *Paratype.* China, Hainan Island, Mt. Jianfeng, 700–800 m, 29 April 2008, ShuChong Bai Co., 1 ♂ (IESNU). China, Hainan Island, Mt. Wuzhi, 800 m, 4 May 2008, Xu-Dong Fei Co., 1 ♂ (IZCAS).
**Remarks.** -- This new species is similar to *L. longitarsis* **n. sp.,** but it differs from the latter in male frontal angle situated behind vibrissal angle in profile; fore tibia without median *p,* mid femur with 2 *p*; the surstyli like a short spoon in profile.
**Etymology.** -- The species name is derived from the Latin “*brev*” meaning short, “*spatula*” meaning spoon, refering to surstyli short spoon-shaped in profile.
**Distribution.** -- China, Hainan Island (Mt. Jianfeng and Mt. Wuzhi).

**Figure 3.**
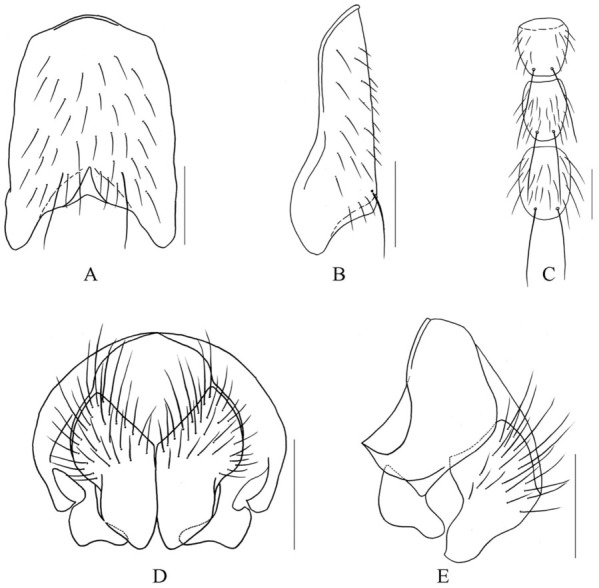
*L. cothurnosurstyla* Xue, Bai et Dong, n. sp.. A. male, sternite 5 in ventral view; B. male, sternite 5 in profile; C. male, sternites 2–4 in ventral view; D. male, Cerci and surstyli in posterior view; E. male, Cerci and surstyli in profile. Scale = 0.2 mm. High quality figures are available online.

***Limnophora cothurnosurstyla* Xue, Bai *et* Dong, n. sp.  (Figure 3 A–E)**
**Holotype Male.** -- Body length 3.6–3.8 mm.
***Head*.** Eye bare; frons about 1/5 of head in width, lateral margins paralleled, with 4 pairs of *ori, 2* pairs of thin *ors,* frontal vitta black, about 4.0 times as wide as fronto-orbital plate, frontal triangle narrow, adjacent to frontal angle, ocellar seta shorter than the the lower *ori,* inner vertical seta long and strong, outer vertical seta about 2/3–4/5 of inner vertical seta in length, upper 2/3 of fronto-orbital plate covered with brown pruinosity, lower 1/3 covered with brown grey pruinosity, parafacial narrow, covered with light grey pruinosity, middle part of parafacial about 1/3 of postpedicel in width; antennae black and long, distal of postpedicel reaching to epistoma almost, basal situated upper 3/5 of eye, postpedicel about 3.0 times as long as wide, arista short ciliated, the longest hair subequal with aristal basal diameter; vibrissal angle at the same vertical line with frontal angle in profile; gena about 1/10 of eye in height, covered with grey pruinosity, genal and postgenal hairs all black, the upper lateral area of the occiput with hairs, prementum shining, about 3.0 times as long as wide; labella small, the length subequal with prementum height, palpus black, slightly longer than prementum.
***Thorax*.** Black in ground color, scutum almost black, lateral of anterior of transverse suture covered with gray pruinosity stripe, scutum and dorsal part of scutellum black mostly, only prescutum with 1 narrow pruinosity stripe, posterior of postpronotal lobe and notopleuron covered with dark grey pruinosity; 4 rows of trichoid *prst-acr,* only 1 pair distinct before scutellum, *dc* 2+4, *ia* 0+2, *pra* seta absent, lateral of scutellum bare, basisternum of prosternum with hairs, notopleural, proepisternum hollow in centre, anepimeron, meron and katepimeron all bare; proepisternal setae 2, upper anterior anepisternal seta 1, katepisternal setae 1+2, the lower one only about 1/3 of the upper one, anterior and posterior spiracles small and brown.
***Wing*.** Transparent slightly, veins brown, basicosta black, costal spine small or absent, distal of Sc slightly straight, dorsal and ventral surface of rascial node with setae, calypters yellowish, lower calypter protrude to tongueshaped; halter yellow.
***Legs*.** Entirely black; fore tibia without median *p;* mid femur without *av,* basal 1/2 with setiform *a* row, 2 super-medial *a* long and strong, 2 pre-apical *pd, 2* bassal *pv,* mid tibia with 2 median *p;* hind femur with 2–3 *av* in apical 1/3, without *pv,* hind tibia without *av,* with 1 medial *ad,* without *pv;* all tarsi longer than tibia, pulvilli small, about half of claw in length, claw about 3/5 of 5th tarsomere in length.
***Abdomen*.** Black in ground color, oviformshaped almost, covered with dark gray to brownish-gray pruinosity, tergites 3 and 4 with a pair of big black lateral spots respectively, the widest part of spot wider than the length of tergite, tergite 5 with 1 wide median patch, syntergite 1+2 all black except middle part, tergite 5 only discal scutellar setae and posterior marginal scutellar setae distinctly, tergite 6 and sternite 1 bare, posterior margin of sternites 2 to 4 with a pair of long setae respectively. Sternites 2–5, cerci and surstyli as in Figure 3 A–E.
**Female.** — Unknown.
**Type material.** -- *Holotype.* China, Hainan Island, Mt. Wuzhi, 800 m, 3 May 2008, ShuChong Bai Co., ♂ (IESNU). ***Paratype*.** China, Hainan Island, Mt. Wuzhi, 800 m, 3 May 2008, Shu-Chong Bai Co., 1 ♂ (IESNU). China, Hainan Island, Mt. Jianfeng, 700–800 m, 30 April 2008, Xu-Dong Fei Co., 2 ♂♂ (IZCAS).
**Remarks.** -- This new species is similar to *L. malailei* Emden, 1965, but it differs from the latter in male frons about 1/5 of head in width, frontal vitta black; lateral of anterior of transverse suture covered with gray pruinosity stripe; calypters yellowish; hind femur with 2 bassal *pv,* hind femur with 2–3 *av* in apical 1/3, hind tibia without *av*; abdomen oviformshaped.
**Etymology.** -- The species name is derived from the Greek words “*cothurn*” meaning boot, “*surstylus*” meaning surstyli, refering to the surstyli boot-shaped in posterior view.
**Distribution.** -- China, Hainan Island (Mt. Jianfeng and Mt. Wuzhi).

**Figure 4.**
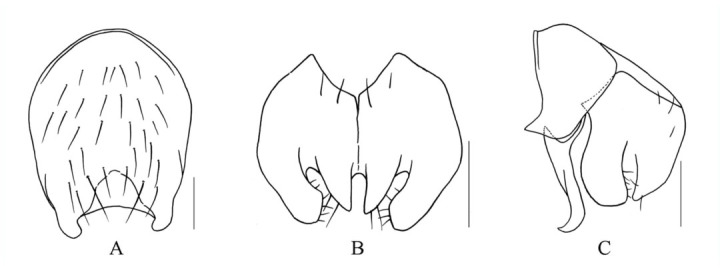
*L. dyadocerca* Xue, Bai et Dong, n. sp.. A. male, sternite 5 in ventral view; B. male, Cerci and surstyli in posterior view; C. male, Cerci and surstyli in profile. Scale = 0.2 mm. High quality figures are available online.

***Limnophora dyadocerca* Xue, Bai *et* Dong, n. sp.  (Figure 4A–C)**
**Holotype Male.** -- Body length 2.0–2.2 mm.
***Head*.** Eye bare; frons black, upper part about 0.52 times as wide as head, lower part about 0.43 times as wide as head, with 2 pairs of *ori*, almost in the same length, with 1 forewards hair in its upper, with 1 pairs of *ors*, a short and proclinate hair always in the lower part, upper part of frontal vitta about 8.0 times as wide as fronto-orbital plate, frontal triangle unobvious, reaching to upper 1/3 of *ors* at most; outer vertical seta about half of inner vertical seta, inner vertical seta slightly longer than ocellar seta, ocellar seta slightly longer than the *ori,* fronto-orbital plate covered with dark brown pruinosity, parafacial covered with light grey pruinosity, the width of parafacial about half of postpedicel in width; antennae black brown, postpedicel long and broad, about 3.0 times as long as broad, and its width subequal with length of pedicel, basal 1/6–1/5 becoming wide, most aristal hairs subequal with its basal diameter; vibrissal angle situated behind frontal angle in profile; gena covered with dense and dark grey pruinosity, genal hairs black and sparse, genal height about 1/5 of width of postpedicel at most, and about 1/12 of eye height, the upper lateral area of the occiput with hairs, prementum shining, about 2.2–2.5 times as long as wide; labella small, the length subequal with height of prementum, palpus black, slightly longer than prementum.
***Thorax*.** Black in ground color, most part of scutum black, proepisternum covered with dark gray pruinosity, anterior 1/3 of prescutum covered with dark gray pruinosity, and with a black median vitta obviously, posterior 2/5 of postscutum covered with gray pruinosity, and with 3 narrow brown vittae, all vittae reaching to scutoscutellar suture; postpronotal lobe and its posterior part covered with grayish- white pruinosity, scutellum all black; 2 rows of trichoid *prstacr, dc* 2+3, without obvious *ia* and *pra,* basisternum of prosternum with hairs, notopleural, lateral of scutellum, proepisternum hollow in centre, anepimeron, meron and katepimeron all bare; proepisternal setae 2, upper anterior anepisternal seta 1, katepisternal setae 1+2, lower one about 2/5 of anterior one in length, anterior and posterior spiracles small and brown.
***Wing*.** Slightly transparent, veins brown, basicosta black, costal spine absent, dorsal and ventral surface of rascial node with setae, calypters grayish-white, posterior margin of lower calypter dark brown, knob of halter big and black, stalk black brown.
***Legs*.** Entirely black, femur thin and long; fore tibia without median *p*; mid femur without *av,* basal half without distinct *a* row, with 1 preapical *pd*, apical 3/5 with distinct *pv,* mid tibia with 1 median *p*; hind femur with 1 apical *av,* without *pv,* hind tibia with 1 *av,* with 1 medial *ad,* without *pv*; all tarsi longer than tibia, pulvilli small, about half of claw in length, claw about 3/5 of 5th tarsomere in length.
***Abdomen*.** Black in ground color, cone-shaped, syntergite 1+2 black mostly, tergites 3 and 4 with a pair of dark brown triangular spots respectively, tergite 5 with indistinct small lateral spots, middle and lateral of tergites 3 to 5 covered with light gray pruinosity, sternite 1 bare, posterior margin of sternite 5 narrow, cerci split to four forks (Figure 4B), inner two forks pointed, outer two long and broad, distal of surstyli hook-shaped and bend forwards. Sternite 5, cerci and surstyli as in Figure 4A–C.
**Female.** — Unknown.
**Type material.** -- ***Holotype*.** China, Hainan Island, Mt. Wuzhi, 800 m, 2 May 2008, Xu-Dong Fei Co., ♂ (IESNU). ***Paratype*.** China, Hainan Island, Mt. Wuzhi, 800 m, 2 May 2008, Shu-Chong Bai and Xu-Dong Fei Co., 2 ♂♂ (IZCAS). China, Hainan Island, Mt. Jianfeng, 700–800 m, 30 April 2008, Xu-Dong Fei Co., 1 ♂ (IESNU).
**Remarks.** -- This new species is similar to *L. ypocerca* **n. sp.,** but it differs from the latter in male upper part of frontal vitta about 8.0 times as wide as fronto-orbital plate, frons with 2 pairs of *ori*; thorax without obvious *ia*; calypters grayish-white, posterior margin of lower calypter dark brown, knob of halter big and black, stalk black brown; mid femur with 1 pre-apical *pd*; abdomen cone-shaped, cerci split to two parts (four forks), inner two forks pointed, outer two long and broad, distal of surstyli hook-shaped and bend forwards.
**Etymology.**--The species name is derived from the Greek words “*dyad*” meaning two, “*cerca*” meaning cerci, refering to cerci split to two parts.
**Distribution.** -- China, Hainan Island (Mt. Jianfeng and Mt. Wuzhi).

**Figure 5.**
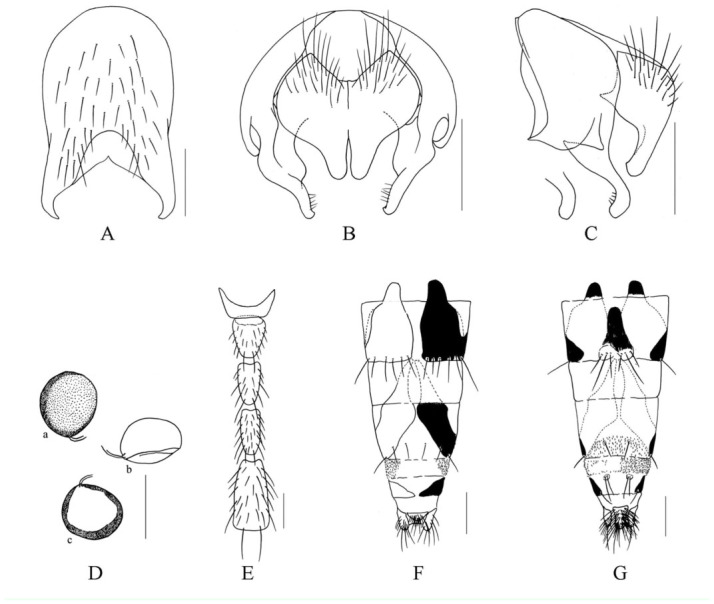
*L. longitarsis* Xue, Bai et Dong, n. sp.. A. male, sternite 5 in ventral view; B. male, Cerci and surstyli in posterior view; C. male, Cerci and surstyli in profile. D. female, a. spermatheca in sclerotized view, b. spermatheca in profile, c. spermatheca in membranous view; E. male, sternites 2–4 in ventral view; F. female, ovipositor in dorsal view; G. female, ovipositor in ventral view. Scale = 0.2 mm. High quality figures are available online.

***Limnophora longitarsis* Xue, Bai *et* Dong, n. sp.  (Figure 5A–G)**
**Holotype Male.** -- Body length 3.5–3.8 mm.
*Head.* Eye bare; frons about 1.2–1.3 times as wide as postpedicel, about 1/10–1/9 of head in width, frontal vitta black, about 2.0 times as wide as fronto-orbital plate, lower 3/5 of frons with 5–6 pairs of intilted *ori*, lower 2/5 with 3 pairs of thin *ors,* the ocellar seta longer than the longest *ori,* upper part of fronto-orbital plate covered with brownish-gray pruinosity, the lower part and parafacial covered with grayish-white pruinosity, parafacial about 2/5 of postpedicel in width; antennae black, postpedicel about 3.0 times as long as wide, arista short ciliated, the longest hair subequal with aristal basal diameter; lunule brown, vibrissal angle situated behind frontal angle in profile, gena about 1/8 of eye in height, genal and postgena hairs all black, the upper lateral area of the occiput with black hairs; prementum shining, about 3.0 times as long as wide; labella small, the length subequal with prementum height, palpus black, slightly longer than prementum.
***Thorax*.** Black in ground color, proepisternum covered with gray pruinosity distinctly, scutum black mostly, lateral of anterior of transverse suture to notopleuron covered with gray pruinosity, anterior part of scutoscutellar suture covered with brown-gray pruinosity, anterior 3/4 of postscutum with black transverse stripe, posterior margin of transverse stripe extending to back, but not reaching to scutoscutellar suture; 4 rows of trichoid *prst-acr*, only 1 pair distinct before scutellum, *dc* 2+3, *ia* 0+1, *pra* hairlike, notopleural, proepisternum hollow in centre, anepimeron, meron and katepimeron all bare, basisternum of prosternum with hairs, scutellum all black, lateral and ventral surface bare, upper anterior anepisternal seta 0–1, proepisternal setae 2, upper anterior anepisternal seta 1, katepisternal setae 1+1.
***Wing*.** Transparent slightly, veins brown, basicosta black, costal spine small and short, distal of Sc slightly straight, dorsal and ventral surface of R_4+5_ vein in basal part and radial node all with setae, calypters yellowish, lower calypter projecting and tongue-shaped, halter yellow.
***Legs*.** Entirely black. Fore tibia with 1 median *p,* tartus of front legs about 1.8–1.9 times as long as fore tibia; mid femur without *av,* basal 2/5 with setiform *a* row (one of them large on super-medial), 2 pre-apical *pd,* without obvious *pv,* ventral surface with a long seta basally, mid tibia with 1 median *p*; distal 1/3 of hind femur with 3–4 *av,* without *pv,* hind tibia with 1 median *av,* 1 super-medial *ad,* without *pv.* Mid and hind tarsi longer than tibia, fore pulvilli subequal with claws in length, mid and hind pulvilli shorter than claws, claws about 1/2 of 5^th^ tarsomere in length.
***Abdomen*.** Black in ground color, similar to oviform-shaped, covered with brownish-grey pruinosity, tergites 3 and 4 with a pair of triangular lateral spots respectively, the distance between the lateral spots about 1/3 of the length of tergite, the widest part of spot wider than the length of tergite, tergite 5 with a trapeziform median spot indistinctly, syntergite 1+2 black except lateral of posterior part with brownish-grey pruinosity, sternite 1 bare. Sternites 2–5, cerci and surstyli as in Figure 5A–C.
**Female.** -- Frons about 1/3 of head in width, with 4 pairs of intilted *ori*, outer with small setae, with 2 pairs of *ors,* frontal triangle reaching to front of frons, frontal vitta about 4.0 times as wide as fronto-orbital plate, fronto-orbital plate and parafacial covered with gray white pruinosity, parafacial about 2/5 of postpedicel in width, vibrissal angle at the same vertical line with frontal angle in profile; *ia* 0+1(2); tartus of front legs about 1.6–1.7 times as long as fore tibia; tergites 3 and 4 with a pair of triangular lateral spots, the distance between the lateral spots about 1/5 of the length of tergite, syntergite 1+2 almost black, a pair of tergites 6 and 7 respectively, all long and strong, posterior margin of tergite 6 wide, with a gap in middle of tergite 6, but not cloven, sternites 7 and 8 divide into 2 parts, sternite 7 with a seta, tergites 6 to 8 extend to ventral surface, tergite 8 with 2 setae, hypoproct with many dense and long setae, cerci broad and large, 3 spermathecas, surface 1/3 transparent and membranous, but not sunken. Spermatheca and ovipositor as in Figure 5D, F–G. The other characters are the same as male.
**Type material. -- *Holotype*.** China, Hainan Island, Mt. Jianfeng, 700–800 m, 29 April 2008, Shu-Chong Bai Co., ♂ (IESNU). ***Paratype*.** China, Hainan Island, Mt. Jianfeng, 700–800 m, 13 September 2007, Jing Du Co., 1 ♂ (IESNU); 29 April 2008, ShuChong Bai Co., 2 ♂♂, 3 ♀♀ (IESNU), 2 ♂♂, 2 ♀♀ (IZCAS); 30 April 2008, Shu-Chong Bai and Xu-Dong Fei Co., 6 ♂♂, 6 ♀♀ (IESNU), 6 ♂♂, 5 ♀♀ (IZCAS).
**Remarks.** -- This new species is similar to *L. albonigra* Emden, 1965, but it differs from the latter in male parafacial about 2/5 of postpedicel in width; thorax with 4 rows of *prst-acr,* calypters yellowish, tartus of front legs about 1.8–1.9 times as long as fore tibia, mid femur without distinct *pv,* hind tibia with 1 median *av,* hind femur with 3–4 *av,* without *pv;* syntergite 1+2 black except lateral of posterior part with brownish-grey pruinosity.
**Etymology.** -- The species name is derived from the Latin words “*long*” meaning long and “*tarsis*” meaning tartus, referring to tartus of front legs long, about 1.8–1.9 times as long as fore tibia.
**Distribution.** -- China, Hainan Island (Mt. Jianfeng).

**Figure 6.**
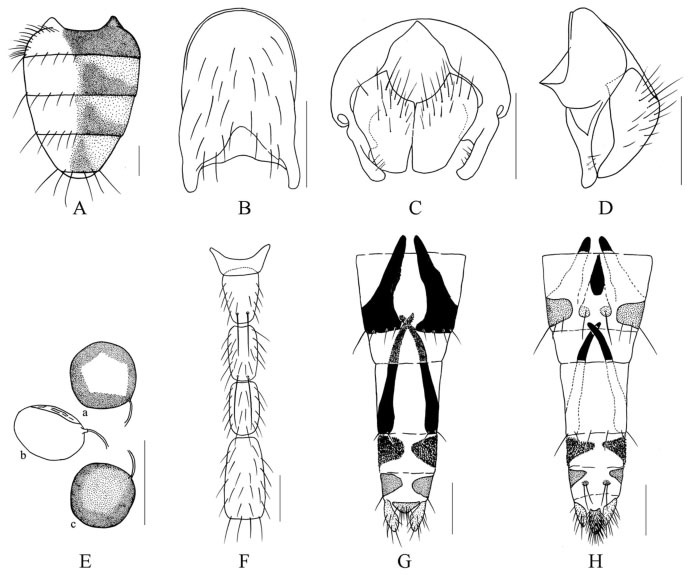
*L. nuditibia* Xue, Bai et Dong, n. sp.. A. male, abdomen in profile; B. male, sternite 5 in ventral view; C. male, Cerci and surstyli in posterior view; D. male, Cerci and surstyli in profile; E. female, a. spermatheca in membranous view, b. spermatheca in profile, c. spermatheca in sclerotized view; F. male, sternites 2–4 in ventral view; G. female, ovipositor in dorsal view; H. female, ovipositor in ventral view. Scale = 0.2 mm. High quality figures are available online.

***Limnophora nuditibia* Xue, Bai *et* Dong, n. sp.  (Figure 6A–H)**
**Holotype Male.** -- Body length 2.5–2.7 mm.
***Head*.** Eye bare; frons about 2/5 of head in width, with 2 pairs of intilted *ori*, upper 2/5 with 2 pairs of upper *ors,* frontal vitta black, about 5.0 times as wide as fronto-orbital plate, frontal triangle shining, reaching to frontage of frons, ocellar seta longer than the longest *ori,* fronto-orbital plate and parafacial covered with dark gray pruinosity, lower part of parafacial became narrow obviously, parafacial about 1/3 of postpedicel in width, which only wirelike in profile; antennae black, wide and large, distal of postpedicel reaching to epistoma almost, postpedicel about 3.0 times as long as wide, arista short pubescent, the longest hair subequal with aristal basal diameter; lunule dark black, vibrissal angle at the same vertical line with frontal angle or slightly situated behind in profile; gena about 1/8 of eye in height, genal and postgena hairs all black, the upper lateral area of the occiput with black hairs, prementum shining, about 2.5 times as long as wide; labella small, the length subequal with prementum height, palpus black, slightly longer than prementum.
***Thorax*.** Black in ground color, proepisternum covered with caesious pruinosity distinctly, scutum black mostly, lateral of anterior of transverse suture covered with narrow and gray pruinosity stripe, posterior of postpronotal lobe and notopleuron covered with light grey pruinosity, anterior 4/5 of postscutum with black transverse stripe, posterior margin of transverse stripe extending to back, but not reaching to scutoscutellar suture; 2–3 rows of trichoid *prst-acr*, only 1 pair distinct before scutellum, *dc* 2+3, *ia* 0+2, *pra* absent, basisternum of prosternum with hairs, notopleural, proepisternum hollow in centre, anepimeron, meron and katepimeron all bare, scutellum all black, lateral and ventral surface bare; upper anterior anepisternal seta 1, proepisternal setae 2, upper anterior anepisternal setae 2, katepisternal setae 1+2, the lower one only about 1/3–2/5 of the upper one.
***Wing*.** Transparent, veins brown, basicosta black, costal spine small and short, distal of Sc slightly straight, dorsal and ventral surface of rascial node with setae, upper calypter yellowish, lower calypter white and protrude to tongue-shaped; halter yellow.
***Legs*.** Entirely black; fore tibia without median *p*; mid femur without *av,* basal part with setiform *a* row, the super-medial one strong, with 1–2 pre-basal *pv,* 2 pre-apical *pd,* mid tibia with 1 super-medial *p;* hind femur with 2 *av* in apical 1/4, with 1 super-medial *pv* and 1 sub-basal *pv,* hind tibia without *av,* with 1 super-medial *ad,* without *pv;* each tarsi longer than tibia, pulvilli and claw small, shorter than half of 5th tarsomere.
***Abdomen*.** Abdomen as in Figure 6A. Black in ground color, short oviform-shaped, covered with brownish-gray pruinosity, tergites 3 and 4 with a pair of short and flat triangular lateral spots respectively, dark black, the width of spot about 2.0 times as long as the tergite, tergite 5 with triangular median spot, dark brown, syntergite 1+2 all black, sternite 1 bare. Sternites 1–5, cerci and surstyli as in Figure 6B–D, F.
**Female.** -- Body length 2.8–3.2 mm. Mid femur with 0–1 pre-basal *pv,* hind femur with 1 apical *av;* abdomen oviform-shaped, tergite 5 with a median stripe, a pair of tergites 6, tergite 7 thin and long, crossed apically, posterior margin of sternite 6 wide, cloven in middle part, sternites 6 and 8 divide into 2 parts, sternite 7 disappeared, tergite s6 to 8 extend to ventral surface, tergite 8 with 2 setae, hypoproct with dense and long hairs, cerci broad and large, 3 spermathecas, surface 1/3 transparent and membranous, but not sunken. Spermatheca and ovipositor as in Figure 6E, G–H. The other characters are the same as male.
**Type material. -- *Holotype*.** China, Hainan Island, Mt. Wuzhi, 700–800 m, 4 may 2008, Shu-Chong Bai Co., ♂ (IESNU). ***Paratype*.** China, Hainan Island, Mt. Bawang, 700 m, 14 September 2007, Shu-Chong Bai Co., 1 ♀ (IESNU). China, Hainan Island, Mt. Jianfeng, 700–800 m, 11 September 2007, Dandan Zhao and Jing Du Co., 3 ♂♂*,* 1 ♀ (IESNU); 13 September 2007, Dan-Dan Zhao, Jing Du and Shu-Chong Bai Co., 1 ♂, 2 ♀♀ (IESNU), 2 ♀♀ (IZCAS); 29 April 2008, Shu-Chong Bai Co., 3 ♂ (IESNU); 30April 2008, ShuChong Bai and Xu-Dong Fei Co., 5 ♂♂, 1 ♀ (IESNU), 6 ♂♂ (IZCAS). China, Hainan Island, Mt. Wuzhi, 800 m, 19 September 2007, Shu-Chong Bai Co., 1 ♀ (IESNU); 2 May 2008, Shu-Chong Bai and Xu-Dong Fei Co., 3 ♂♂, 4 ♀♀ (IESNU), 2 ♂♂, 4 ♀♀ (IZCAS); 3 May 2008, Shu-Chong Bai and Xu-Dong Fei Co., 3 ♂♂, 3 ♀♀ (IESNU), 3 ♂♂, 3 ♀♀ (IZCAS); 4 May 2008, Shu-Chong Bai and Xu-Dong Fei Co., 14 ♂♂, 3 ♀♀ (IESNU), 13 ♂♂, 3 ♀♀ (IZCAS).
**Remarks.** -- This new species is similar to *L. subplumosa* Emden, 1965, but it differs from the latter in male frontal vitta about 5.0 times as wide as fronto-orbital plate, parafacial about 1/3 of postpedicel in width, arista short pubescent; hind tibia without *av.*
**Etymology.** -- The species name is derived from the Latin “*mid*” meaning bare and “*tibia*” meaning tibia, refering to hind tibia of the male without *av.*
**Distribution.** -- China, Hainan Island (Mt. Bawang, Mt. Jianfeng and Mt. Wuzhi).

**Figure 7.**
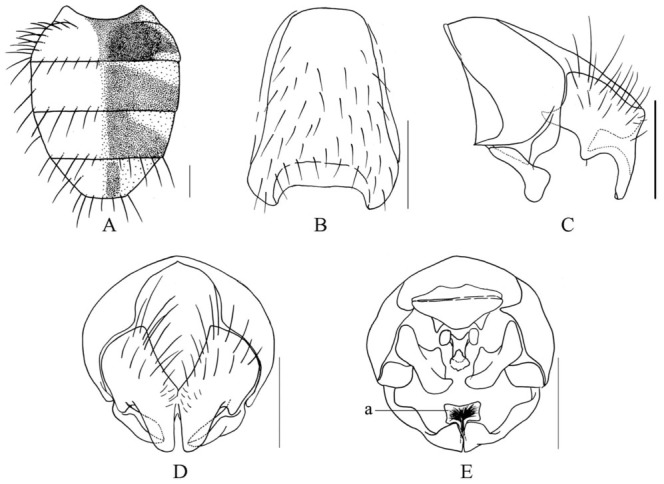
*L. ypocerca* Xue, Bai et Dong, n. sp.. A. male, abdomen in profile; B. male, sternite 5 in ventral view; C. male, Cerci and surstyli in profile; D. male, Cerci and surstyli in posterior view; E. male, Cerci and surstyli in ventral view, a. the small subcercal structure. Scale = 0.2 mm. High quality figures are available online.

***Limnophora ypocerca* Xue, Bai *et* Dong, n. sp.  (Figure 7A–E)**
**Holotype Male.** -- Body length 2.8–3.0 mm.
***Head*.** Eye bare; frons about 4/9–1/2 of head in width, with 3–4 pairs of small and short *ori,* 1 pair of retroverted *ors,* frontal vitta black, about 3.5 times as wide as fronto-orbital plate, covered with dark brown pruinosity, frontal triangle reaching to anterior 3/4 of frons, ocellar seta about 2.0 times as long as the longest *ori,* posterior vertical seta long and strong, about 4.0 times as long as the longest *ori,* outer vertical seta short and small, slightly longer than postocellar seta, fronto-orbital plate and parafacial covered with grey pruinosity, parafacial about half of postpedicel in width; antennae black, postpedicel about 3.0 times as long as wide, about 2.5 times as long as pedicel, basal 1/3 of arista becoming wide, aristal hairs slightly short, mostly hairs longer than aristal basal diameter; vibrissal angle situated behind frontal angle in profile; gena covered with caesious pruinosity, genal hairs black and sparse, genal height about 2/7 of eye height, the upper lateral area of the occiput with hairs, proboscis thin and long, prementum shining, about 3.5 times as long as wide; labella small, the length subequal with height of prementum, palpus thin and long, only distal part slightly intumescent, and covered with light grey pruinosity distally, subequal with prementum in length.
***Thorax*.** Black in ground color, proepisternum covered with caesious pruinosity, scutum dark brown mostly, postpronotal lobe and its posterior part, notopleuron, postalar callus, anterior and posterior margins of scutoscutellar suture all covered with light grey pruinosity, inner margin of *prst-dc* rows with a pair of narrow and caesious pruinosity vittae; 2–3 rows of trichoid *prst-acr,* out of order, *dc* 2+3, *ia* 0+2, without obvious *pra,* basisternum of prosternum with hairs, notopleural, lateral of scutellum, proepisternum hollow in centre, anepimeron, meron and katepimeron all bare; proepisternal setae 2, upper anterior anepisternal seta 1, katepisternal setae 1+2, lower one about half of anterior one, anterior and posterior spiracles small and brown.
***Wing*.** Slightly transparent, veins brown, basicosta black, costal spine small or absent, dorsal and ventral surface of rascial node with setae, calypters yellowish, lower calypter protrude to tongue-shaped; halter yellow.
***Legs*.** Entirely black, but all coxae, trochanters and femora covered with caesious pruinosity; fore tibia without median *p*; mid femur without *av,* basal half with setiform *a* row, with 2 pre-apical *pd,* 1–2 basal *pv,* mid tibia with 1 median *p,* apical *v* long and strong; hind femur with 1–2 pre-apical *av,* without *pv,* hind tibia with 1 submedial *av,* 1 medial *ad,* without *pv*; all tarsi longer than tibia, pulvilli small, about half of claw in length, claw about 3/5 of 5th tarsomere in length.
***Abdomen*.** Abdomen as in Figure 7A. Black in ground color, wide and oviform-shaped, covered with gray to light gray pruinosity, tergites 3 to 5 with narrow and light median vitta, tergites 3 and 4 with broad and dark brown spots respectively, tergite 5 with long strip-shaped lateral spots, sternite 1 bare, posterior margin of sternite 5 wide, without long seta, distal of surstyli intumescent in profile, cerci with an small subcercal structure hidden behind the distal half of cereal plate (Figure 7C-a). Sternite 5, cerci and surstyli as in Figure 7B–E.
**Female.** -- Unknown.
**Type material. -- *Holotype*.** China, Hainan Island, Mt. Jianfeng, 700–800 m, 30 April 2008, Shu-Chong Bai Co., ♂ (IESNU). *Paratype.* Same data as holotype, 1 ♀ (IZCAS).
**Remarks. --** This new species is similar to *L. rufimana* Strobl, 1893, but it differs from the latter in male frons about 4/9–1/2 of head in width; thorax with 2–3 trichoid *prst-acr* rows; mid femur without *av,* mid tibia with 1 median *p*; distal of surstyli intumescent in profile, a small subcercal structure hidden behind the distal half of cereal plate.
**Etymology. --** The species name is derived from the Greek words “*ypo-*” meaning sub-, “*cerca*” meaning cerci, refering to distal half of cereal plate with a small subcercal structure.
**Distribution. --** China, Hainan Island (Mt. Jianfeng).
